# Long-term efficacy of a printed or a Web-based tailored physical activity intervention among older adults

**DOI:** 10.1186/1479-5868-10-104

**Published:** 2013-09-02

**Authors:** Denise Astrid Peels, Catherine Bolman, Rianne Henrica Johanna Golsteijn, Hein de Vries, Aart Nicolaas Mudde, Maartje Marieke van Stralen, Lilian Lechner

**Affiliations:** 1 Department of Psychology, Open University of the Netherlands, PO Box 2960, 6401, DL Heerlen, the Netherlands; 2 Department of Health Promotion, Maastricht University, Maastricht University Medical Centre, PO Box 616, 6200, MD Maastricht, the Netherlands; 3 Care and Public Health Research institute (Caphri), Maastricht University, Maastricht, the Netherlands; 4 EMGO Institute for Health and Care Research and the Department of Public and Occupational Health, VU University Medical Centre, van der Boechorststraat 7, 1081, BT Amsterdam, the Netherlands

**Keywords:** Tailored intervention, Physical activity, Effect, Implementation costs, Older adults, Print-delivered, Web-based

## Abstract

**Background:**

This study provides insight into the long-term efficacy (i.e. 12 month results) of the Web-based or print-delivered tailored Active Plus intervention (with and without environmental approach) to promote physical activity (PA) among the over-fifties. Differences in effect among subgroups are studied as well.

**Methods:**

Intervention groups (i.e. print-delivered basic (PB; *N* = 439), print-delivered environmental (PE; *N* = 435), Web-based basic (WB; *N* = 423), Web-based environmental (WE; *N* = 432)) and a waiting list control group (*N* = 411) were studied in a clustered randomized controlled trial. Intervention participants received tailored advice three times within 4 months. Long-term effects (12 months after the intervention has started, i.e. 8 months after the intervention was completed) on PA (i.e. self-reported weekly minutes and days of sufficient PA) were tested using multilevel linear regression analyses. Participants’ age, gender, BMI, educational level, PA intention and the presence of a chronic physical limitation were considered to be potential moderators of the effect.

**Results:**

Overall, the Active Plus intervention was effective in increasing weekly days of sufficient PA (*B*=0.49; *p*=.005), but ineffective in increasing weekly minutes of PA (*B*=84.59; *p*=.071). Per intervention condition analysis showed that the PB-intervention (*B*_days_=0.64; *p*=.002; *B*_min_=111.36; *p*=.017) and the PE-intervention (*B*_days_=0.70; *p*=.001; *B*_min_=157.41; *p*=.001) were effective in increasing days and minutes of PA. Neither Web-based conditions significantly increased PA, while the control group decreased their PA. In contrast to the intervention effect on minutes of PA, the effect on weekly days of PA was significantly moderated by the participants’ baseline intention to be sufficiently physically active.

**Conclusions:**

In general, after 12 months the print-delivered interventions resulted in stronger effects than the Web-based interventions. The participants’ baseline intention was the only significant moderator of the intervention effect. All other assessed user characteristics did not significantly moderate the effect of the intervention, which might indicate that the intervention is sufficiently tailored to the different participant characteristics. Additional efforts should be taken to increase the sustainability of Web-based interventions.

**Trial registration:**

Dutch Trial Register: NTR2297.

## Background

Regular physical activity (PA) reduces the risks on multiple health problems which often become more prevalent when people age [1-3]. Furthermore, regular PA enables older adults to maintain their mobility and independence, and to improve muscle strength, cognitive functioning, and emotional well-being, and it may thereby improve quality of life [1,4-7]. Because of the aging population in the Netherlands, stimulating PA among people over 50 years of age is of major relevance.

To stimulate PA among people aged over 50, the Active Plus intervention was developed following the Intervention Mapping protocol [8,9]. The Active Plus intervention is a computer-tailored intervention, which includes the provision of tailored advice on three occasions delivered during four months (i.e. immediate advice after baseline measurement, a second advice two months after baseline, and a third advice within four months after baseline based on a second assessment). The intervention can be delivered by a printed and in a Web-based version [9]. Furthermore, the intervention provides an opportunity to include additional environmental components (e.g. advice on local PA possibilities), intended to positively change people’s perceptions of the PA possibilities in their own environment [8]. The Active Plus intervention was thus available in four versions, namely: (1) a basic printed intervention, targeting PA awareness, psychosocial determinants and PA self-regulation components; (2) an environmental printed intervention, targeting environmental determinants in addition to the basic intervention; (3) a basic Web-based intervention; and (4) an environmental Web-based intervention.

The four intervention conditions were compared in a clustered randomized controlled trial (RCT). Participants were recruited via direct mailing in communities of the Municipal Health Council regions participating in this project. Per region, one intervention condition was implemented to obtain the most realistic estimates since in a real-life setting most likely only one of the intervention conditions is implemented. Baseline results showed that the print-delivered intervention resulted in a higher reach (19% of the people who were invited agreed to participate) than the Web-based intervention (12% agreed to participate), and that dropout during the intervention was higher in the Web-based intervention groups (53%) than in the print-delivered intervention groups (39%) [10]. Six months after baseline (i.e. two months after completing the intervention), the Active Plus intervention was proven to be effective when compared to the waiting list control group. Intervention participants increased their PA on average by about 250 minutes per week [11] (Peels DA, Van Stralen MM, Bolman C, Golsteijn RHJ, De Vries H, Mudde AN, Lechner L: The differentiated effectiveness of a printed versus a Web-based tailored intervention to promote physical activity among the over-fifties, Submitted), while the control group increased its PA behaviour by 49 weekly minutes. Only the printed interventions were effective in increasing the weekly days of sufficient PA behaviour (at least 30 minutes of moderate physical activity).

For future implementation of the intervention, more insight is needed into the long-term efficacy of the four intervention conditions. As a programme evaluation showed that the printed intervention was more used (i.e. 92.7-98.2% read, 70.1-76.5% kept, and 39.9-56.8% discussed) than the Web-based intervention (i.e. 55.1-86.8% read, 39.6-56.3% kept, and 16.9-32.0% discussed), and the additional environmental intervention components were more used than the basic intervention components [12], the long-term efficacy might differ between the intervention conditions as well.

Previous studies showed that certain participant characteristics were related to the reach, attrition and short-term effects of the Active Plus intervention; Web-based intervention participants were significantly younger, more often men, had a higher BMI and a lower PA intention than participants of the printed intervention [10]; participants with a low PA intention were more likely to dropout [10]; and in women and among older participants, the printed intervention resulted in stronger short-term intervention effects than the Web-based intervention (Peels DA, Van Stralen MM, Bolman C, Golsteijn RHJ, De Vries H, Mudde AN, Lechner L: The differentiated effectiveness of a printed versus a Web-based tailored intervention to promote physical activity among the over-fifties, Submitted). Additional research should reveal whether the long-term efficacy of the intervention is also dependent on these participant characteristics, since this might have important implications for the large-scale implementation of the intervention.

The current study provides insight into (1) the long-term efficacy of the intervention conditions (12 months after the intervention has started, i.e. 8 months after the intervention was completed); and (2) the differences in long-term efficacy among subgroups.

## Methods

For the purpose of the study, a 5-arm clustered Randomized Controlled Trial was conducted, which was registered at the Dutch Trial Register (NTR2297) and approved by the Medical Ethics Committee of Atrium–Orbis–Zuyd (MEC 10-N-36).

### Study design

The intervention groups and the waiting list control group (who received no intervention until the end of the study period) were studied in a clustered randomized control trial (RCT). There were evaluation assessments (i.e. questionnaires) at the start (T0: also the basis for the first and second tailored advice), 3 months after baseline (T1: also the basis for the third tailored advice), 6 months after baseline (T2) and 12 months after baseline (T3) [9]. For the purpose of this study only the T0 and T3 measure were used.

### Participants and procedures

**Figure 1:**
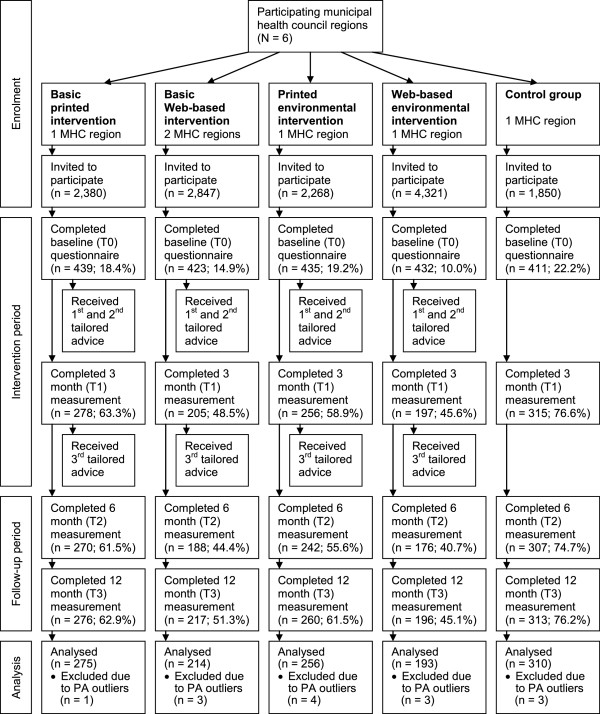
**Flow diagram of the selection, enrolment and participation of respondents.** Note: Percentages are reported in contrast to the number of baseline participants.

Participants (Dutch speaking adults aged over fifty) were recruited via direct mailing in communities of the Municipal Health Council regions (MHC; N = 6) participating in this project. Communities were matched on their urbanity, percentage of people with a low SES, percentage of people with a high SES, percentage of immigrants, and the percentage of people aged over 50. The regions were randomly assigned to one of the five research arms: (1) basic printed intervention; (2) environmental printed intervention; (3) basic Web-based intervention; (4) environmental Web-based intervention; or (5) control group. Participants had to be over 50 years of age (no maximum age), and need to have sufficient understanding of the Dutch language. No other in- or exclusion criteria were set. Each MHC provided a list of addresses of a random sample of eligible participants living in the selected matched communities. Figure 1 provides an overview of the number of invitations that were distributed in order to reach an even number of participants aged over 50 at baseline for each condition, and of the number of participants at the enrolment and participation stages. A power calculation (effect size = 0.4, power = 80%, intracluster correlation coefficient = .1) showed that at the baseline (T0) about 420 participants were needed for each intervention condition to include a minimum of 250 participants per research condition at the 12-month (T3) assessment, bearing in mind an expected dropout rate of 40% during the 1-year follow-up based on a previous project [13]. Participants were included from November 2010 until March 2011 [10]. Invitations for the printed intervention contained an information letter, a questionnaire, a prepaid return envelope and a form for giving informed consent. Invitations to the Web-based intervention contained a similar information letter, additional information about how to fill in a Web-based questionnaire and a personal username and password to log on to the Active Plus website. For the online intervention group, informed consent was administered online.

### Intervention

The Active Plus intervention is a systematically developed computer tailored, theory- and evidence-based intervention to stimulate PA among people aged over fifty [8,9]. The intervention aimed to influence awareness, initiation and maintenance of PA by targeting pre-motivational constructs (i.e. awareness, knowledge), motivational constructs (i.e. attitude, self-efficacy, social influence, intrinsic motivation and intention) and post-motivational constructs (i.e. commitment, strategic planning, self-regulation skills, action planning and coping planning) [8].

Intervention participants received advice on three occasions, tailored to the answers they gave in previous assessments [8,9]: (1) after the baseline assessment (immediate advice for the Web-based intervention and within two weeks for the printed intervention); (2) two months after the baseline assessment; and (3) within four months after baseline assessment feedback on progress was given based on the second assessment (again, immediate advice for the Web-based intervention and within two weeks for the printed intervention, after completing the second questionnaire). Participants in the Web-based intervention received the invitations for follow-up assessment by email, including a link to the Web-based questionnaire. Participants in the printed intervention received the invitation for follow-up assessment by mail, together with the follow-up questionnaire and a prepaid return envelope.

The intervention provides general information about the benefits of PA especially for older adults, such as the importance of physical activity to maintain a healthy cognitive state. The specific content of the basic tailored intervention advice depended on the participants’ personal characteristics (e.g. age, gender and educational level) and psychosocial characteristics (e.g. pre-motivational, motivational and post-motivational constructs as described above), and PA behaviour, and the extent to which they were planning to change their behaviour (i.e. every participant received all the tailored information, but the participants PA behaviour and stage of change determined in which advice the information was provided [8]). For example, contemplators received feedback in the first advice on their PA behaviour to raise awareness of their own physical inactivity, followed by feedback on their intention to become more physically active and on their perceived pros, cons and self-efficacy (with special attention to the most important PA barriers in this age group). Participants were stimulated using role model stories and to write down their own intrinsic PA motivation. In the second advice, contemplators received advice about their perceived social support, they received practical information about physical activity possibilities and they were encouraged to formulate action plans. The third tailored advice provided feedback about the progress in behaviour and determinant scores in the previous months [8]. The intervention with additional environmental components contained the same information as the basic tailored intervention, with additional tailored advice on local possibilities and initiatives for being physically active (e.g. walking or cycling routes in their own neighbourhood) [8]. The content of the Web-based and printed intervention were identical; however, interactive components were integrated into the Web-based version such as the use of videos instead of pictures, and Google neighbourhood maps in stead of printed neighbourhood maps [9].

Participants in the Web-based intervention received, in addition to the Web-based advice, an email with a copy (pdf-format) of their personal advice that was in the same format as the printed version. The tailored advice contained between five and eleven pages of text and illustrations, depending on (changes in) PA behaviour and determinant scores.

The different intervention conditions result in different implementation costs as well. Due to the lower reach of the Web-based intervention than of the printed intervention (12% vs. 19% response), recruitment costs for the Web-based intervention are higher than for the printed intervention. On the other hand, Web-based interventions result in lower handling, printing and postage costs. Furthermore, the provision of additional environmental information results in higher costs.

### Measures

#### Physical activity behaviour

Total weekly days of sufficient PA and minutes of moderate to vigorous PA were assessed using the validated self-administered Dutch Short Questionnaire to Assess Health Enhancing Physical Activity (SQUASH) [14]. Total weekly days of sufficient PA was measured by a single item: ‘On how many days per week are you, in total, moderately physically active by undertaking, for example, brisk walking, cycling, chores, gardening, sports, or other physical activities for at least 30 minutes?’. Total weekly minutes of moderate to vigorous PA was calculated by multiplying the frequency (how many days per week), and duration (how many hours and minutes per day) of leisure and transport walking, leisure and transport cycling, sports, gardening, household chores and odd jobs performed with moderate or vigorous intensity. The reproducibility (*r*_spearman_ = 0.58; 95% CI = 0.36–0.74) and relative validity (*r*_spearman_ = 0.45; 95% CI = 0.17–0.66) of the SQUASH are reasonable for the general adult population [14]. A study of Wagenmakers et al. showed that using the SQUASH in an older population can be considered as a fairly reliable tool and that the validity was comparable to those of other questionnaires [15].

#### Moderators

Age, gender (0 = *men*; 1 = *women*), educational level, BMI, intention to be sufficiently physically active and the presence of a chronic physical limitation (0 = *absent*; 1 = *present*) were selected as potential moderators of the intervention effect, and were all assessed in the baseline questionnaire. Educational level was categorised as ‘low’ (primary, basic vocational or lower general school) or ‘high’ (higher general secondary education, preparatory academic education, medium vocational school, higher vocational school or university), according to the Dutch educational system. BMI was calculated by dividing self-reported weight by height in metres squared. Participants were classified as being underweight (BMI < 18.5 kg/m^2^), healthy weight (BMI 18.5–24.9 kg/m^2^) or overweight (BMI > 25 kg/m^2^). The participants’ intention to be sufficiently physically active was assessed using three items on a 10-point scale (e.g. ‘To what degree do you intend to be sufficiently physically active?’: 1 = *absolutely not*; 10 = *absolutely sure*). The average sum score of the three variables was used in the analyses (Cronbach’s α = 0.938).

Other variables were also assessed, but are not described here as they are not related to the current focus of this study.

### Statistical analyses

#### Baseline and dropout characteristics

One-way analyses of variance (ANOVAs) and Chi-square tests were conducted to test for baseline differences in participant characteristics (i.e. age, gender, BMI, educational level, intention to be sufficiently physically active and the presence of chronic physical limitation) among the five research conditions. These characteristics are considered as covariates in the remaining analyses. Hierarchical logistic regression analyses were performed to study whether these baseline characteristics were predictors of dropout at the 12 month measurement, and whether these predictors differed between the research conditions. Analyses were performed using SPSS for Windows (Version 18).

#### Effect on physical activity behaviour

Participants were nested within neighbourhoods resulting in the probability of interdependence between them. To account for this interdependence, multilevel linear regression analyses were performed with a random intercept for two levels (1: individual; 2: neighbourhood). Each 12 month outcome (i.e. total weekly days of sufficient PA and weekly minutes of moderate to vigorous PA) was first regressed onto the Active Plus intervention in general (i.e. the four intervention conditions together, in contrast to the control group), and secondly regressed onto the dummies for the different research conditions independently (with the control group as a reference case), its baseline values and the covariates (gender, age, education, intervention type, BMI, intention, having a chronic limitation). The analyses were repeated with different intervention conditions as a reference case, to study the difference between the intervention conditions. Furthermore, it was studied whether the intervention effect was moderated by the aforementioned participant characteristics. This moderation effect was studied by adding an interaction term to the model between these participant characteristics and the dummies for the intervention conditions.

By regressing the outcome values against their baseline values, the amount of change in the variables is represented independently of their baseline value. This method is preferred to using absolute change scores because groups with lower levels are more likely to increase their levels by chance than groups with higher levels [16]. Analyses were applied to the total dataset, including missing data. Applying multilevel analyses to an incomplete dataset has been shown to give more accurate estimations than applying imputation methods [17]. Respondents were excluded from the analyses when they reported being physically active for more than 6,720 minutes per week since being physically active for 7 days per week, over 16 hours per day was considered to be impossible [14].

To obtain a better interpretation of the intervention effects and a better comparison with other tailored interventions, Cohen’s *d* effect sizes (ESs) were calculated for each intervention condition compared to the control group. ESs were defined as the mean differences in effect between the intervention conditions and the control group (corrected for baseline PA) divided by the pooled standard deviations for those means, in which d = 0.15, 0.20, and 0.25 are considered for small, medium and large effects [18].

Additionally, all analyses were repeated with the inclusion of all participants who filled in the baseline measurement, using the multiple imputation method (based on the participants’ age, gender, baseline intention, and PA behaviour at T0, T1, T2 and T3; 5 times imputed) for participants who did not fill in the 12 months questionnaire. Several studies have shown that the multiple imputation method is preferable over single-imputation methods, such as last observation carried forward [19].

## Results

### Study population

An overview of the number of respondents who filled in the questionnaires is shown in Figure 1. A total of 1,262 persons filled in the 12-month questionnaire (59% response), of which 14 participants were excluded from further analyses because they reported being physically active for more than 6,720 minutes per week at either the baseline or 12-month measurement. Attrition analyses showed that participants in Web-based conditions (*B* = .614, *p* < .001), participants who received additional environmental information (*B* = .398, *p* < .001), younger participants (*B* = −.016, *p =* .006) and participants with a lower intention to be physically active (*B* = −.121, *p* < .001) were more likely to dropout from the 12 month follow-up measurement. Predictors of dropout did not differ between the intervention conditions.

Table 1 shows the baseline characteristics of the participants who were included in the 12-month analyses (complete cases) and the significance of the overall difference between the groups. The control group (*p* = .008) was significantly older than the Web-based environmental intervention group. Although no differences were found between the groups in the total minutes of PA performed per week, in the Web-based basic intervention group, more people already complied with the PA norm at baseline than in the control group (*p* = .002), the printed environmental group (*p* = .020) and the Web-based environmental intervention group (*p* = .007). This difference can be explained by the fact that, as compliance with the norm is a combination of the minutes of PA and the days of PA per week (minimum of 30 minutes a day, 5 days a week moderate to intensive PA), the Web-based basic intervention group performed significantly more days of PA per week at baseline than the control group (*p* = .000), the printed environmental group (*p* = .029) and the Web-based environmental intervention group (*p* = .010). Furthermore, a significant difference was found between the research conditions in the number of participants who have a chronic physical limitation. Within the Web-based basic and within the Web-based environmental intervention condition, fewer respondents (p < .001) reported having a chronic physical limitation than in the control group and in the printed conditions. No other significant differences were found between the intervention groups. Remaining analyses were corrected for all assessed baseline characteristics.

**Table 1:** Baseline socio-demographic characteristics of the intervention groups and the control group included at the 12-month measurement

	**Printed basic**	**Printed env.**	**Web-based basic**	**Web-based env.**	**Control group**	**Sign. diff.**
	**(n = 275)**	**(n = 256)**	**(n = 214)**	**(n = 193)**	**(n = 310)**	
Gender (% men)	47.8	43.7	52.8	51.8	49.0	.305
Age (years)	63.2	63.7	62.6	61.6	64.1	**.011**
(Mean ± SD)	(± 8.3)	(± 8.9)	(± 7.2)	(± 7.8)	(± 9.0)	
BMI (kg/m^2^)						.345
Underweight (%)	1.5	2.0	0.5	0.5	2.3	
Healthy (%)	50.7	46.6	42.6	47.6	43.6	
Overweight (%)	47.8	51.4	56.9	51.8	54.1	
Education (% low)	41.5	49.0	43.5	47.4	49.5	.257
Having a chronic physical limitation (%)	55.6	50.4	29.9	31.6	52.3	**.000**
Physical activity (PA)						
Minutes of PA (per week)	742.0	744.5	741.7	721.2	788.4	.848
(Mean ± SD)	(± 637.2)	(± 624.1)	(± 637.2)	(± 754.9)	(± 718.6)	
Days of PA (per week)	4.2	4.0	4.6	3.9	3.8	**.001**
(Mean ± SD)	(± 1.9)	(± 2.0)	(± 2.1)	(± 1.9)	(± 2.0)	
Compliance with the PA norm (%)	44.5	41.4	51.4	38.9	38.2	**.028**
Intention to be sufficiently	7.8	7.9	7.7	7.6	7.6	.200
Physically active (mean ± SD)	(± 1.5)	(± 1.5)	(± 1.4)	(± 1.5)	(± 1.6)	

NB: The bold numbers reflect a significant difference of p<.05.

### Intervention effect on physical activity

Overall, the Active Plus intervention (i.e. provision of the four intervention conditions) was effective in increasing weekly days of sufficient PA (*B* = 0.49; *p* = .005; ES = .18), but ineffective in increasing weekly minutes of PA (*B* = 84.59; *p* = .071; ES = .20). More in-depth analyses into the different Active Plus intervention conditions (see Table 2), showed that both the printed basic intervention (*B* = 111.36; *p* = .017; ES = .21) and the printed environmental intervention (*B* = 157.41; *p* = .001; ES = .32) were significantly effective in increasing total minutes of PA when compared to the control group. The weekly minutes of PA increased on average 57 minutes in the printed basic intervention group and 114 minutes in the printed environmental intervention group, whereas the control group showed a decrease of 58 minutes. Neither of the Web-based interventions resulted in a significant increase in total minutes of PA (there was an average decrease of 3 minutes in the Web-based basic intervention, and a decrease of 9 minutes in the Web-based environmental intervention). In both the printed and the Web-based intervention group, no significant difference in the intervention effect was observed between the basic and the environmental condition on total minutes moderate to vigorous PA behaviour. Furthermore, the effect of the intervention on the total minutes of PA (assessed for each intervention condition separately) was not significantly moderated by the assessed user characteristics (i.e. age, gender, BMI, educational level, PA intention and the presence of a chronic physical limitation), although a borderline significant (*p* = .066) interaction was found between the basic printed intervention condition and the participants baseline intention.

**Table 2:** Intervention effect after 12 months (overall and per intervention condition)

**Complete cases**							**Imputed data**				
	***n***	**B**	**SE**	***p***	**95% confidence interval**	**ES**	**B**	**SE**	***p***	**95% confidence interval**	**ES**
Effect on minutes PA											
Overall	907	84.59	43.29	.071	−8.22 – 117.40	.20	94.04	36.79	**.012**	21.32–166.76	.22
PB	264	111.36	46.45	**.017**	19.83 – 202.89	.21	102.01	43.82	**.021**	15.48–188.54	.21
PE	235	157.41	48.04	**.001**	63.15 – 251.66	.32	132.27	42.46	**.002**	48.88–215.67	.30
WB	214	32.11	49.69	.581	−65.37 – 129.61	.12	73.32	47.74	.131	−22.54–169.20	.22
WE	193	20.33	51.13	.691	−79.98 – 120.64	.10	64.69	48.18	.186	−32.16–161.55	.15
Effect on days of PA											
Overall	884	0.49	0.15	**.005**	0.16 – 0.81	.18	0.36	0.11	**.001**	0.15–0.58	.12
PB	252	0.64	0.17	**.002**	0.28 – 1.00	.23	0.54	0.12	**.000**	0.29–0.78	.21
PE	228	0.70	0.17	**.001**	0.33 – 1.07	.38	0.51	0.12	**.000**	0.28–0.75	.29
WB	211	0.21	0.18	.238	−0.16 – 0.59	-.07	0.19	0.12	.127	−0.05–0.43	-.06
WE	193	0.31	0.18	.112	−0.08 – 0.69	.14	0.19	0.13	.160	−0.07–0.44	.03

NB: Results are presented in contrast to the control group (n_minutes_ = 283; n_days_ = 269); *PB* printed basic, *PE* printed environment, *WB* Web-based basic, *WE* Web-based environment. The bold numbers reflect a significant intervention effect (p<.05).

Both the printed basic intervention (*B* = 0.64; *p* = .002; ES = .23) and the printed environmental intervention (*B* = 0.70; *p* = .001; ES = .38) were also significantly effective in increasing total days of PA compared to the control group. The printed basic intervention group increased its days of PA behaviour from 4.2 days to 4.8 days, and the printed environmental intervention group increased its days of PA behaviour from 4.0 days to 4.9 days. No significant effects on days of PA behaviour were found in the Web-based interventions. The total days for the basic Web-based intervention group remained equal, and the environmental Web-based intervention resulted in a not significant increase from 3.9 to 4.4 days, whereas the control group increased its PA days from 3.8 to 4.0. No significant difference in the intervention effect was observed between the basic and the environmental condition on weekly days of sufficient PA, in neither the printed nor the Web-based intervention.

In the printed environmental intervention condition, the effect of the intervention on the total days of PA per week was moderated by the participants’ baseline intention to be physically active (*B* = 0.20; SE = 0.10; *p* = .038). The printed environmental intervention condition was only significantly effective (*B* = 0.81; SE = 0.19; *p* = .000; ES = .45) in increasing the weekly days of PA behaviour in participants with a high baseline intention to be physically active (≥ 5 on a scale from 1–10). In the other intervention conditions, no interaction effect by baseline intention was observed. All other assessed user characteristics (i.e. age, gender, BMI, educational level and the presence of a chronic physical limitation) did not significantly moderate the effect of the intervention on the total days of PA., although a borderline significant interaction (*p* = .052) was found between the basic Web-based intervention condition and the participants BMI.

In the analyses applied to the imputed dataset, the same relations were found to be significant as in the complete case analyses regarding the effects of the individual intervention conditions on PA behaviour (see Table 2). However, whereas the intervention in total (the four intervention conditions together) was shown to be insignificant in the complete case analyses regarding the effect on minutes of PA (*p* = .071), a significant relation (*p* = .012) was found in the imputed dataset. Analyses applied to the imputed dataset resulted in stronger effect sizes regarding the effect of both Web-based interventions on the weekly minutes of PA when compared to the analyses applied to the complete cases. Effect sizes regarding the effects on the weekly days of PA became smaller. Furthermore, no significant interaction terms were found in the analyses applied to the imputed dataset, indicating that the moderation effect of the participants’ baseline intention on the intervention effect on days of PA has disappeared.

## Discussion

This study provides insight in the long-term efficacy of four intervention conditions (i.e. printed basic, printed environmental, Web-based basic and the Web-based environmental) of the Active Plus PA intervention in (subgroups of) adults aged over fifty. These insights provide indications for the feasibility of the large-scale implementation of printed or online tailored PA interventions (with or without an environmental approach) among older adults.

Overall, when considering only the complete cases, the Active Plus intervention as a whole was effective in increasing weekly days of sufficient PA (ES = .18), but only borderline effective in increasing weekly minutes of PA (ES = .20; *p* = .071). When performing similar analyses on a dataset in which the outcome measures for participants who dropped-out during follow-up were imputed, the Active Plus intervention was found to be significantly effective on both outcome measures. By imputing the missing data power was increased, resulting in a significant overall intervention effect on minutes of PA.

More in-depth analyses showed that 12 months after the intervention started, only the printed conditions resulted in significantly increased weekly minutes and days of PA. As short-term results (i.e. 6 months after the intervention started) showed that also the Web-based conditions were able to increase the weekly minutes of PA (Peels DA, Van Stralen MM, Bolman C, Golsteijn RHJ, De Vries H, Mudde AN, Lechner L: The differentiated effectiveness of a printed versus a Web-based tailored intervention to promote physical activity among the over-fifties, Submitted), this indicates that the effects of the Web-based conditions are less well maintained than the effects of the printed conditions. Effect sizes of the printed interventions regarding the effect on weekly minutes of PA decreased when follow-up time increased, but less sharply than the effect sizes of the Web-based interventions. Our results are in line with recent meta-analyses [20,21] which both showed decreased intervention effects when follow-up time increased. In contrast to our study, in the meta-analyses no difference in intervention effects between different delivery modes was found (i.e. print, computer, telephone, etc.).

Maintenance (i.e. sustainability) of behavioural intervention effects is of major importance to achieve an impact on public health [22]. More insight into factors that can explain or stimulate the maintenance of interventions is therefore of major relevance. The Active Plus programme evaluation [12] showed that the Web-based intervention materials were less often used, less often saved and less well appreciated than the printed intervention materials. Increasing the appreciation and usability of the Web-based intervention materials might increase the sustainability of the Web-based interventions. Evidence [23,24] suggests that intervention features such as provisions for peer and counsellor support, email contact with supervisors and regular website updates, were related to increased exposure in internet-delivered health behaviour interventions. These features can be considered as intervention boosters. The current Active Plus website only provided participants with the tailored advice, none of the suggested intervention features were implemented in the website. Since research has identified a clear dose–response relationship between the intensity of the intervention and the resulting behaviour change, it can be expected that an increase in website engagement is an important factor for the long-term effectiveness of the Web-based intervention [21]. Integrating the suggested intervention features [23] might help stimulate the sustainability of the intervention.

No statistical significant difference in the intervention effect was observed between the basic and the environmental conditions, in neither the printed nor the Web-based intervention. In practice, however, the difference in effect between the basic and the environmental condition (i.e. of about one hour per week when comparing the effect of the printed basic intervention condition to the printed environmental intervention condition) might still have implications for both health and for policymaking.

The effect of the printed environmental condition (which was the intervention condition in which the participants received the most information) was moderated by the participants’ PA intention; weekly days of PA only increased in participants with a high baseline intention. Possibly only people with a high intention are willing to use all additional environmental information that is provided to them. Participants with a low intention might perceive this intervention as an information overload, resulting in less effect. The intervention effect was not moderated by the participants’ age, gender, BMI, SES, or the presence of a chronic physical limitation. These equal effects in all subgroups might indicate that the intervention was successfully and sufficiently tailored to the different relevant characteristics of the participant, as tailoring is supposed to do.

Whereas our measurements relied on self-reported data through validated questionnaires, the responses can be biased by social desirability. Although self-reports may be less accurate than objective observations, self-administered questionnaires are the most commonly used, and most inexpensive method to use in large-scale studies. Validating intervention effects with objective measurements in the future would be recommendable.

Attrition analyses showed that participants in the Web-based and in the environmental conditions were more likely to dropout from the 12-month assessment. A possible explanation for the higher drop-out within the Web-based conditions might be that it requires more planning to fill in a Web-based questionnaire (i.e. print-delivered questionnaires can be filled in anywhere at any time, while filling in online questionnaires restricts one to a computer). Furthermore, in a printed questionnaire it is easier to resume filling in the questionnaire after pausing, rather than resuming a Web-based questionnaire, due to loading times and additional log-ins [10]. One possible explanation for higher drop-out in the environmental conditions could be (as shown in focus group interviews among intervention participants (Peels DA, Golsteijn RHJ, Lechner L: Advice report on the feasibility of the Active Plus intervention: in depth analyses from focus group interviews with participants, Unpublished)) that the environmental intervention components motivated participants to search for additional information outside of the Active Plus intervention. This might make returning to the Active Plus website less necessary for these participants. Another explanation could be that receiving both tailored advice and additional environmental information could be experienced as an information overload, resulting in intervention dropout. This possible overload of information might also explain why the environmental information does not result in significantly increased intervention effects.

Furthermore, analyses showed that dropout was higher among younger participants and participants with a low PA intention. Due to this selective dropout, the overall effectiveness of this study might be biased. Longitudinal studies often result in missing data due to dropout during follow-up. Using multilevel regression analyses or using multiple imputation methods are often used methods to handle missing data. In the current study, both methods were applied with comparable results, although there were some differences. Which analyses are the most reliable is a major discussion point. Considering only the complete cases regarding the outcome measures provides the best prediction of parameter estimated, but it also results in a loss of power [17] and the effects of the intervention might be overestimated. Multiple imputations do not really add more information but the sample size is increased and the standard error reduced. Since sufficient power is essential when performing moderation analyses (i.e. subgroup analyses), performing the moderation analyses on an imputed dataset might result in the best estimates. However, analyses applied to the imputed dataset showed that the intervention effect was not moderated by any of the assessed participant characteristics. Several studies concluded that it was not necessary using multiple imputations before performing a multilevel analysis on longitudinal data [19,25]. Based on these studies, we might assume that outcomes from the multilevel analyses without applying multiple imputations could be considered to provide the best estimates for our intervention effects.

In this paper the effect of four different intervention conditions are studied and compared to each other, resulting in multiple testing. By performing multiple tests we were able to show the results of this study in a broader perspective and to give a more nuanced picture of the study results. A disadvantage of multiple testing is an increase of the probability of making a Type I error. A Bonferroni correction, however, assumes that all of the hypothesis tests are statistically independent, which is not the case in the current study. The probability of making a Type I error would be less than Bonferroni assumes, and the Bonferroni would be an over-correction. Therefore, we did not apply a Bonferroni correction to the current study results. However, P-values found in the current study were so strong, that even when correcting for multiple testing, results would still have been significant.

## Conclusion

The Active Plus intervention was able to increase PA behaviour till 1 year after the intervention started. The provision of the printed intervention conditions had a more sustained effect on PA behaviour than the Web-based condition of the Active Plus intervention. Increasing the appreciation and usability of the Web-based intervention materials [12] and the integration of intervention boosters (i.e. regular website updates) might increase the sustainability of the Web-based interventions. Additional research should provide more insight (e.g. by additional mediation or pathway analyses) into factors which can explain and stimulate the maintenance of the intervention, especially the Web-based version.

## Abbreviations

ANOVA: Univariate one-way analyses of variance; BMI: Body mass index (weight (in kg) divided by height (in metres squared)); ICER: Cost-effectiveness ratio; MHC: Municipal health council; PA: Physical activity; SD: Standard deviation; SQUASH: Short questionnaire to assess health enhancing physical activity.

## Competing interests

The authors declare that they have no competing interests.

## Authors’ contributions

DPE wrote the first draft of the manuscript. MVS developed the first (printed) version of the Active Plus interventions. RGO and DPE adapted the interventions and translated the printed intervention into a Web-based intervention. RGO and DPE performed the analyses for this study. The process was supervised by ELE, CBO, HDV and AMU. All authors read, modified and approved the final manuscript.
